# Reduction of *Phytophthora palmivora* plant root infection in weak electric fields

**DOI:** 10.1038/s41598-024-68730-y

**Published:** 2024-08-28

**Authors:** Eleonora Moratto, Zhengxi Tang, Tolga O. Bozkurt, Giovanni Sena

**Affiliations:** Department of Life Sciences, Imperial College London, London, UK

**Keywords:** Plant roots, Oomycetes, Root protection, Plant–pathogen communication, Agritech solutions, Plant physiology, Plant signalling, Fungal host response, Fungal biology

## Abstract

The global food security crisis is partly caused by significant crop losses due to pests and pathogens, leading to economic burdens. *Phytophthora palmivora*, an oomycete pathogen, affects many plantation crops and costs over USD 1 billion each year. Unfortunately, there is currently no prevention plan in place, highlighting the urgent need for an effective solution. *P. palmivora* produces motile zoospores that respond to weak electric fields. Here, we show that external electric fields can be used to reduce root infection in two plant species. We developed two original essays to study the effects of weak electric fields on the interaction between *P. palmivora*’s zoospores and roots of *Arabidopsis thaliana* and *Medicago truncatula.* In the first configuration, a global artificial electric field is set up to induce ionic currents engulfing the plant roots while, in the second configuration, ionic currents are induced only locally and at a distance from the roots. In both cases, we found that weak ionic currents (250–550 μA) are sufficient to reduce zoospore attachment to Arabidopsis and Medicago roots, without affecting plant health. Moreover, we show that the same configurations decrease *P. palmivora* mycelial growth in Medicago roots after 24 h. We conclude that ionic currents can reduce more than one stage of *P. palmivora* root infection in hydroponics. Overall, our findings suggest that weak external electric fields can be used as a sustainable strategy for preventing *P. palmivora* infection, providing innovative prospects for agricultural crop protection.

## Introduction

According to the UN/FAO and WHO, the lack of global food security, or the insufficient availability of safe and nutritious food worldwide is a major crisis^1^. A primary contributor to food scarcity is the continuous reduction in crop yields due to pests and pathogens. They cause up to 40% loss in annual yield worldwide, which costs over USD 220 billion^2^.

One of these pests is *Phytophthora palmivora,* a hemibiotrophic oomycete pathogen endemic to tropical areas^3^. It infects over 200 species of plants and causes up to 90% yield loss at an average annual cost of over USD 1 Billion to plantation crops such as cocoa, coconut, oil palm, papaya, and rubber trees^4,5^. The main symptoms consist of fruit and root rot and no preventative crop protection strategy is currently available^6–8^. Establishing an effective preventative method is therefore urgent.

*P. palmivora* infestation of plantations occurs mostly in the rainy season or during irrigation^3,9^. This is because infection is initiated by motile zoospores that require a liquid substrate to swim toward and attach to plant roots^10^. This is followed by germination and the growth of a mycelium inside the host tissue. The cycle is completed when the oomycete’s fruiting body (sporangium) develops and releases more zoospores^9^. A remarkable characteristic of *P. palmivora* zoospores is their electrotactic behavior when exposed to a weak electric field (EF) the zoospores accumulate at the positive electrode and germination rates increase^11,12^.

Zoospore electrotaxis assumes relevance when considering that plant roots exhibit an endogenous bioelectric field generating external ionic currents between the apical root meristem (more negatively charged) and the proximal elongation zone (more positively charged)^13–19^. Interestingly, plant endogenous bioelectric fields are involved in a variety of biotic interactions (recently reviewed in 20), and it has been shown that *P. palmivora* zoospores preferentially attach to the positively charged zone of cocoa roots^20^.

Here, we propose and test the hypothesis that artificial EFs can be used to perturb the natural bioelectric interaction between zoospores and roots, to divert zoospores away from plant roots, and thus act as a crop protection system. We describe a novel setup to perform and quantify *P. palmivora* root infections in hydroponics in the presence of external EFs. We characterize the effect of different external EF intensities and configurations on different stages of root infection in the plant model systems *Arabidopsis thaliana* and *Medicago truncatula*.

## Results

### A novel quantitative assay to investigate the effects of electric fields on *P. palmivora* root infection in hydroponics

**Figure 1:**
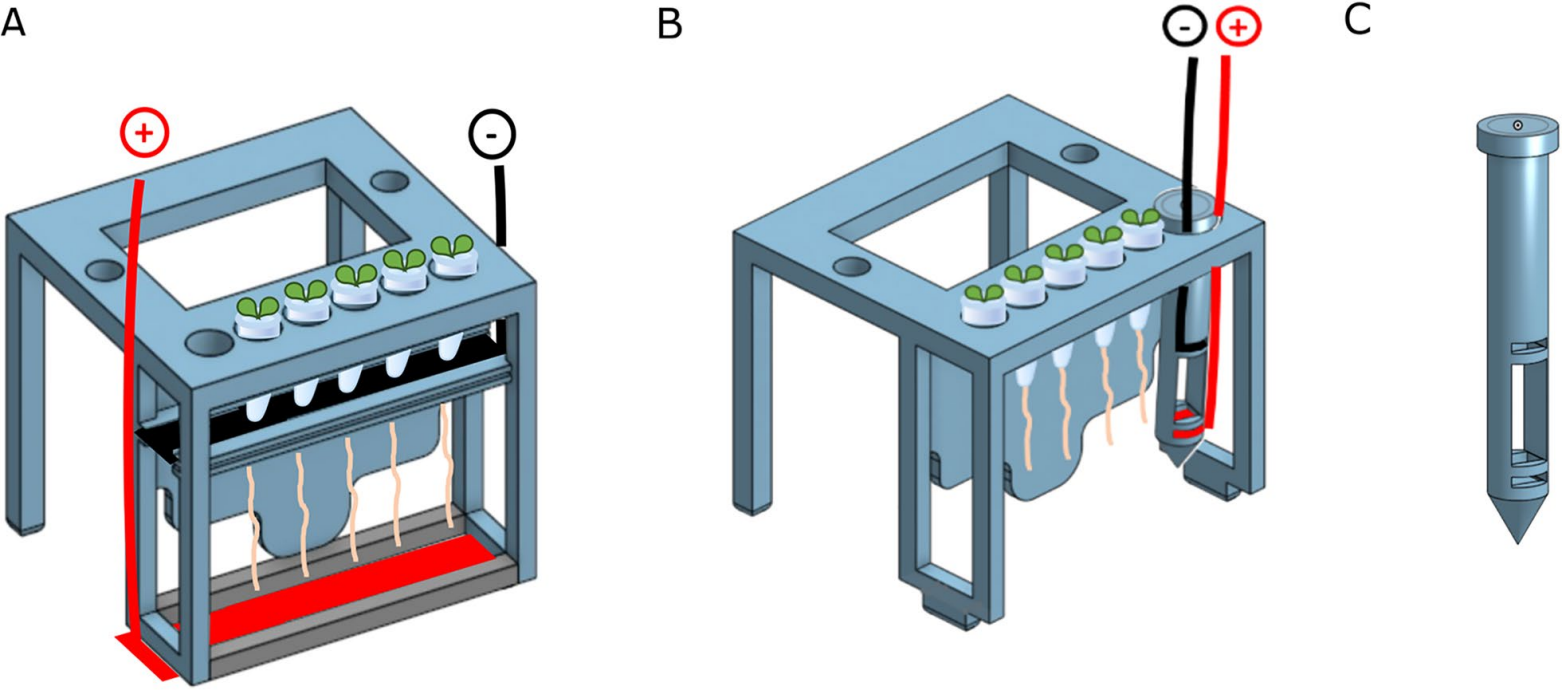
Schematic of the 3D-printed V-box set-up for root infection assays with P. palmivora zoospores in the presence of external electric fields. The negative and positive electrodes are connected to an external power supply. (**A**) Global electric field set-up. The roots are enveloped in a constant ionic current. (**B**) Local electric field set-up. The electrodes are mounted in a mock root located on one side of the V-box, so roots are not enveloped in the ionic current. (**C**) Mock root used to generate the local electric field, with slots used to insert the positive and negative electrodes.

To study the effects of electric field exposure on root infection, we adapted a previously described Voltage-box (V-box) setup^21^ to expose the plant roots to a constant vertical electric field in two configurations: global and local (Fig. 1A,B and Supplementary Fig. 1–4). An exact description of the electric field generated in the liquid medium is complicated by the double layer formed at the electrode’s surface and is beyond the scope of this paper; instead, we will simply refer to the nominal field that would be generated in vacuum (i.e. the ratio between the potential difference and the distance between the electrodes) and to the actual current measured in the circuit (Table 1). Roots were grown vertically in a transparent chamber containing 2 l of a buffered liquid medium.

**Table 1:** Average electrical currents and current density measured for each global nominal electric field applied. s.e.m., standard error of the mean.

Nominal EF (V/cm)	Current (mean ± s.e.m.)	Current density (mean ± s.e.m.)
0.5 V/cm	134 ± 20 μA	0.2 ± 0.03 μA/mm^2^
0.7 V/cm	253 ± 53 μA	0.4 ± 0.09 μA/mm^2^
1.0 V/cm	309 ± 63 μA	0.5 ± 0.10 μA/mm^2^

For the global EF configuration, two long foil electrodes were immersed in the medium and placed underneath (positive) and above the roots (negative) (Fig. 1A, Supplementary Fig. 1 and Supplementary Fig. 2).

For the local EF configuration, we designed and 3D printed a cylindrical support (called “mock root”) with two short foil electrodes (Fig. 1B,C and Supplementary Fig. 3). The mock root (Fig. 1C) is slotted in the V-box next to the plant roots (Fig. 1B and Supplementary Fig. 4).

In both cases, the electrodes are connected to a power supply to generate an electric field and an ionic current parallel to the growing roots (Fig. 1 and Methods). After the EF was turned on, zoospores were pipetted in the center of the top of the V-box, and plantlets were collected for imaging or mRNA extraction and RT-qPCR after 2 h or 24 h infection, respectively.

### Exposure to a global electric field reduces zoospore attachment to *Arabidopsis* roots

**Figure 2:**
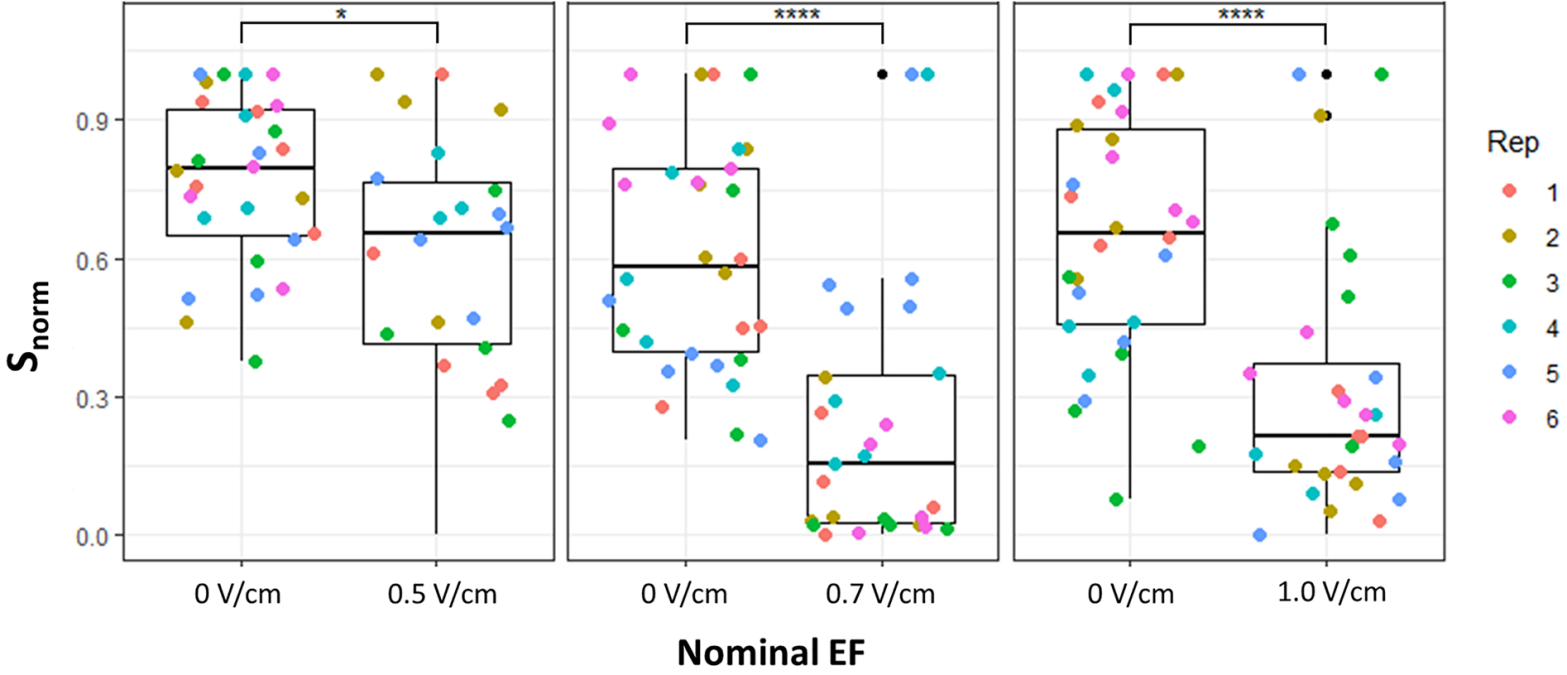
Zoospore attachment to Arabidopsis roots is reduced by exposure to the global electric field. Distributions of the number of zoospores on the surface of roots exposed to 0.5 V/cm, 0.7 V/cm, and 1.0 V/cm in the global configuration. Each point represents ${s}_{norm}$ of one root; each color represents one technical replicate. * = *p*-value < 0.05, **** = *p*-value < 0.0001 ; Wilcoxon test (n = 6 replicates, each with 5 roots).

To test the main hypothesis that an external EF can affect early-stage root infection, we first implemented the global configuration (Fig. 1A) to expose roots to nominal EFs known to cause *P. palmivora* zoospore electrotaxis^22^: 0.5 V/cm, 0.7 V/cm, and 1.0 V/cm, with the corresponding current and current density measured in the system (Table 1). After 2h exposure, when zoospore attachment and germination had occurred^10^, we observed the roots under a confocal microscope and calculated a zoospore-attachment index normalized to take into account variation in total zoospore numbers within each replicate:

$${s}_{norm}= \frac{{s}_{r}}{{s}_{max}}$$

where ${s}_{r}$ is the number of zoospores attached to the root of interest, with $r=\{1;2;3;4;5\}$, and ${s}_{max}$ is the maximum number of zoospores attached to a root within the same technical replicate. We observed a statistically significant decrease in ${s}_{norm}$ with all tested EF intensities (Fig. 2).

### Exposure to a local electric field (mock root) reduces zoospore attachment to *Arabidopsis* roots

**Figure 3:**
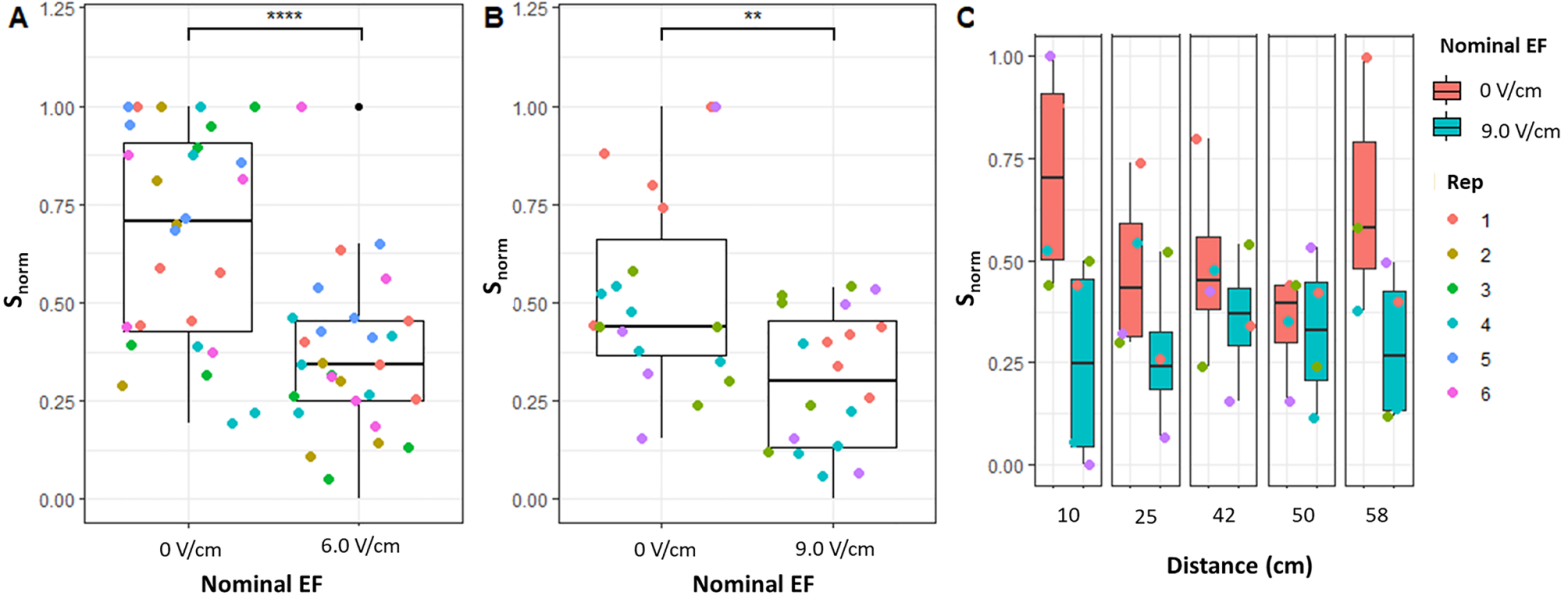
Zoospore attachment to Arabidopsis roots is reduced by exposure to the local electric field. (**A**) Distributions of ${s}_{norm}$ for each Arabidopsis root exposed to ~ 250 µA generated by the local electric field. Each point represents ${s}_{norm}$ for one root; each color represents one technical replicate. **** = *p*-value < 0.0001; Wilcoxon rank sum exact test (n = 6 replicates, each with 6 roots). (**B**) Distributions of ${s}_{norm}$ for each Arabidopsis root exposed to ~ 550 µA generated by the local electric field. Each point represents ${s}_{norm}$ for one root; each color represents one technical replicate. ** = *p*-value < 0.01; Wilcoxon rank sum exact test (n = 4 replicates, each with 6 roots). (**C**) Distributions of ${s}_{norm}$ for Arabidopsis roots exposed to 550 µA generated by the local electric field. The x-axis describes the distance of each plant root from the local electric field (mock root) (n = 4 roots).

We then tested the effect of the local EF configuration (Fig. 1B) on zoospore attachment to Arabidopsis roots, applying 6.0 V/cm to obtain a current ~ 250 µA through the mock root. With this experiment, we aimed to match the lowest best-performing current produced by the global configuration (Fig. 2 and Table 1). As with the global configuration, confocal imaging showed a statistically significant reduction of zoospores attached to roots in the presence of the local EF when compared to the control without EF (Fig. 3A).

Given the local nature of this configuration, we wondered whether the effect on zoospore attachment might depend on the distance between the plant root and the mock root. To test this, we performed the same experiment with a new design that allowed a greater range of distances from the mock root (Supplementary Fig. 5). We observed that, while exposed roots showed an overall reduction in zoospore attachment (Fig. 3B), it was not affected by proximity to the mock root (Fig. 3C).

### Exposure to global electric fields is more efficient than exposure to local ones in reducing zoospore attachment to *Arabidopsis* roots.

**Figure 4:**
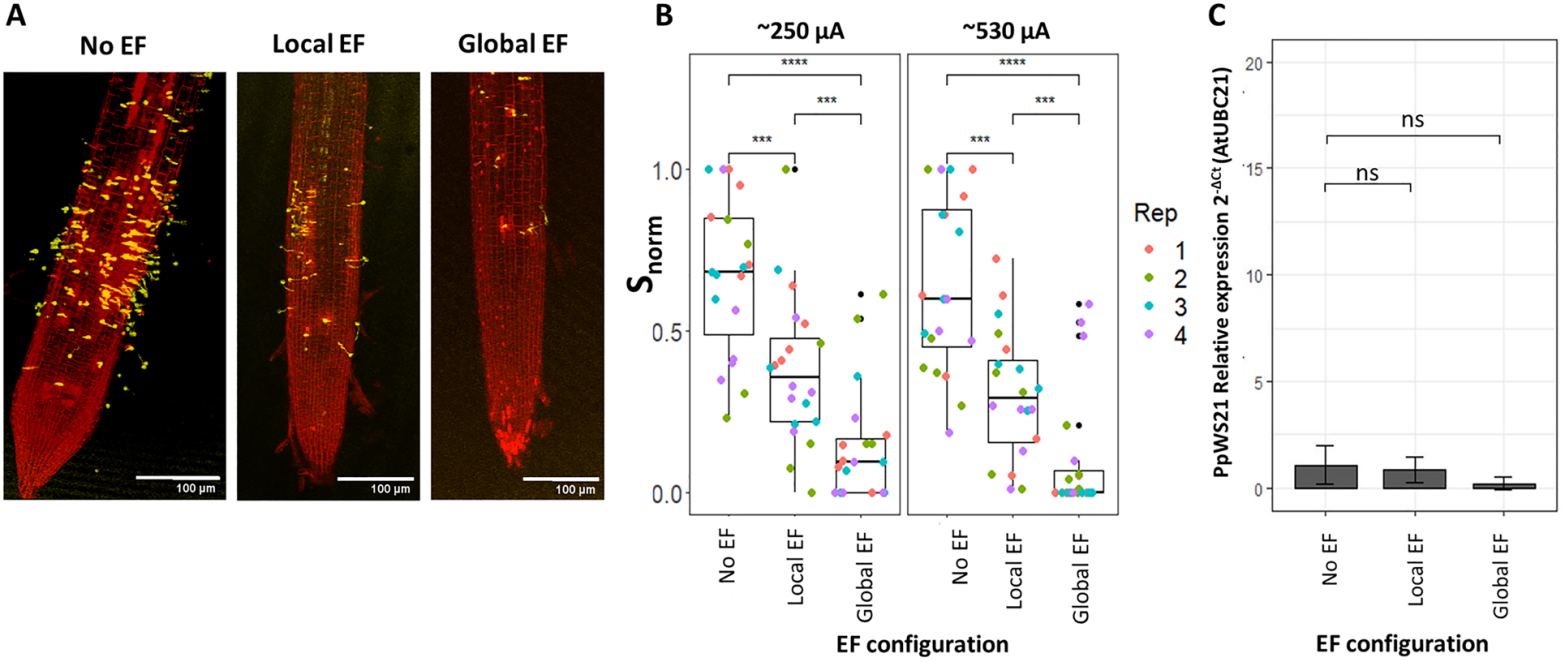
The global EF configuration is more effective than the local (mock root) one in reducing zoospore attachment to Arabidopsis roots. (**A**) Representative confocal images of roots after 2h infection in control conditions or exposed to ~ 250 µA in the local or global configurations. (**B**) A global electric field is more effective at reducing zoospore attachment to Arabidopsis roots regardless of current intensity. Distributions of ${s}_{norm}$ for Arabidopsis roots exposed to ~ 250 µA (left panel) and ~ 530 µA (right panel) in the global and local electric field configurations. Each point represents the ${s}_{norm}$ for one root; each color represents one technical replicate. *** = *p*-value < 0.001, **** = *p*-value < 0.0001; Wilcoxon test (n = 4 replicates, each with 5 roots). (**C**) Pathogen biomass after 24h infection measured as relative expression of a pathogen housekeeping gene (ppWS21) compared to a plant housekeeping gene (atUBC21); each color represents one technical replicate. ns = *p*-value > 0.05; Tukey test (n = 3 replicates, each with 5 roots).

We compared the two configurations using nominal EFs to generate comparable currents of ~ 250 μA and ~ 530 μA (Table 2). In both cases (weak and strong currents) the global EF performed better in reducing zoospore attachment to the roots, as confirmed by confocal imaging (Fig. 4A,B). Interestingly, while the local EF performed about the same at weak and strong currents, the global EF further improved its effect at stronger currents, occasionally achieving zoospore-free roots (Fig. 4B).

**Table 2:** Average electrical currents and current density measured for each global and local nominal electric field applied.

Configuration	Nominal field (V/cm)	Current (mean ± s.e.m.)	Current density (mean ± s.e.m.)
Local EF	6.0 V/cm	222 ± 6 μA	17.4 ± 0.46 μA/mm^2^
Global EF	0.7 V/cm	260 ± 14 μA	0.4 ± 0.02 μA/mm^2^
Local EF	9.0 V/cm	523 ± 42 μA	40.9 ± 3.25 μA/mm^2^
Global EF	1.4 V/cm	536 ± 9 μA	0.86 ± 0.01 μA/mm^2^

Although we were not expecting major *P. palmivora* mycelial growth in the root of Arabidopsis, we asked if a prolonged EF exposure would inhibit *P. palmivora* relative biomass*.* To test this, we exposed the roots to ~ 250 μA in both EF configurations for 24 h, harvested the plant, and extracted mRNA. We quantified the transcripts of the *P. palmivora* housekeeping gene ppWS21 relative to those of the Arabidopsis housekeeping gene atUBC21, a proxy for pathogen biomass^23^. As expected, low *P. palmivora* biomass was detected with or without EF (Fig. 4C), confirming that in Arabidopsis the main effect of EF is on zoospore attachment to the root surface.

### Exposure to local or global EFs has a small effect on primary root growth and no effect on root gravitropism in Arabiddopsis

**Figure 5:**
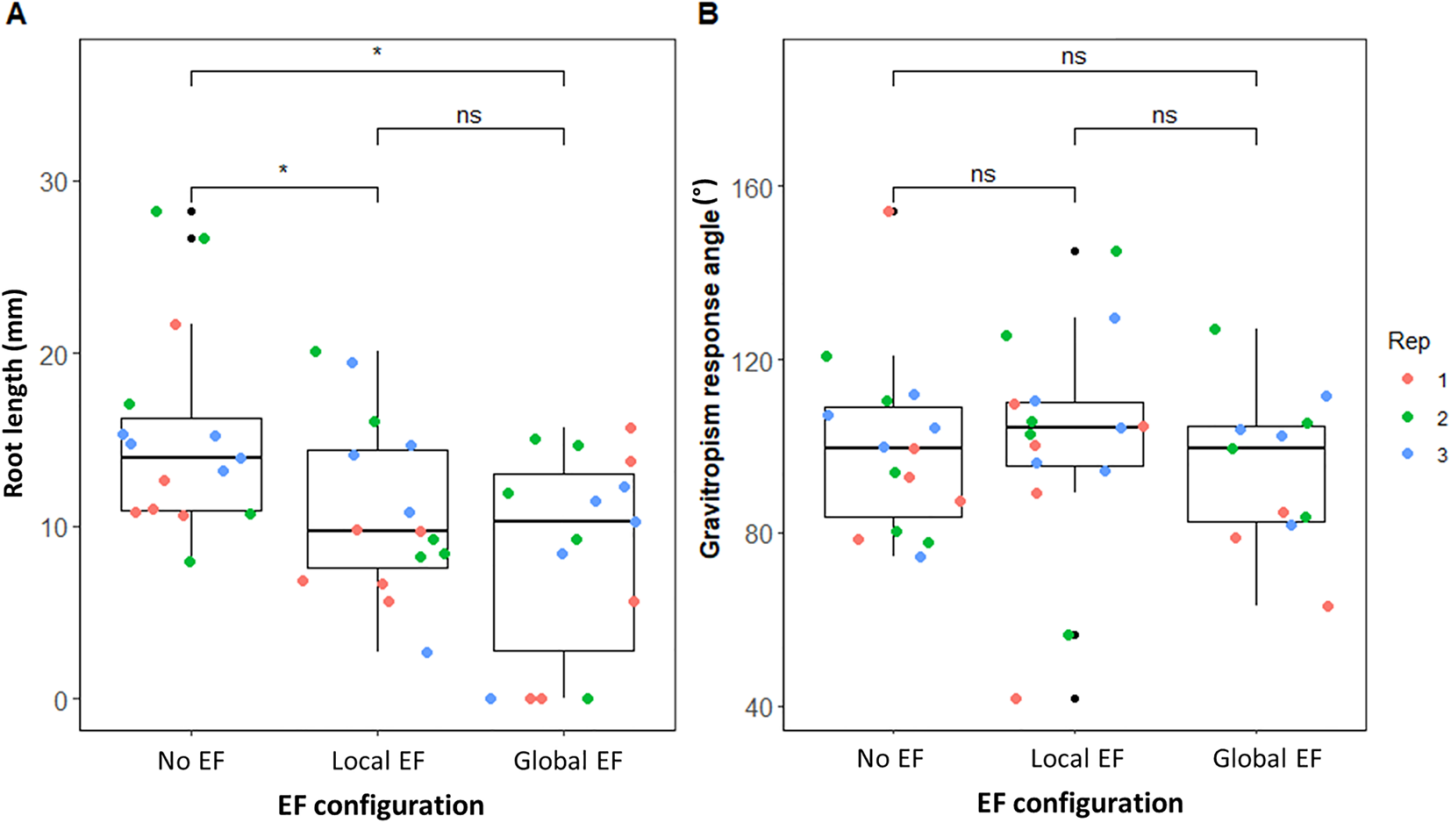
24h exposure of Arabidopsis roots to ~ 250 µA mildly reduces root growth but does not affect its gravitropic response. (**A**) Distribution of root growth of seedlings exposed to ~ 250 µA for 24h. Each point represents the growth in mm of one primary root, each color represents one technical replicate. ns = *p*-value > 0.05, * = *p*-value < 0.05; Wilcoxon rank sum exact test (n = 3 replicates, each with 5 roots). (**B**) Distribution of root gravitropism response angle of seedling exposed to ~ 250 µA and turned 90° relative to gravity for 24h. Each point represents the angle between one primary root and the gravity vector; each color represents one technical replicate. ns = *p*-value > 0.05, * = *p*-value < 0.05; Wilcoxon rank sum exact test (n = 3 replicates, each with 5 roots).

Having demonstrated an effect on *P. palmivora’s* zoospores, we then asked whether exposure to EFs affected plant health. First, we run a limited assessment of Arabidopsis roots by measuring growth and gravitropic response after exposing them for 24 h to ~ 250 μA in the global and local configurations in the V-box (Fig. 5). While we observed a small but statistically significant decrease in root length due to EF exposure (Fig. 5A), no significant effect on gravitropic response was detected (Fig. 5B). We then moved to a more realistic essay on plant health, by exposing Arabidopsis roots to ~ 250 μA in the local EF configuration (mock root) in soil for over 14 days and measuring leaf number and the whole plant’s dry and fresh weights: no statistically significant difference was observed between exposed and not-exposed plants (Supplementary Fig. 6).

Taken together, these results suggest that exposure to ~ 250 μA does not affect overall plant biomass or the shoot and only has a minimal effect on the primary root.

### External EFs reduce zoospore attachment and mycelial growth in *Medicago* truncatula roots

To quantify the efficiency of our setup with multiple plant species, we tested it with *Medicago truncatula*, a popular model system susceptible to *P. palmivora* root infection^24^.

**Figure 6:**
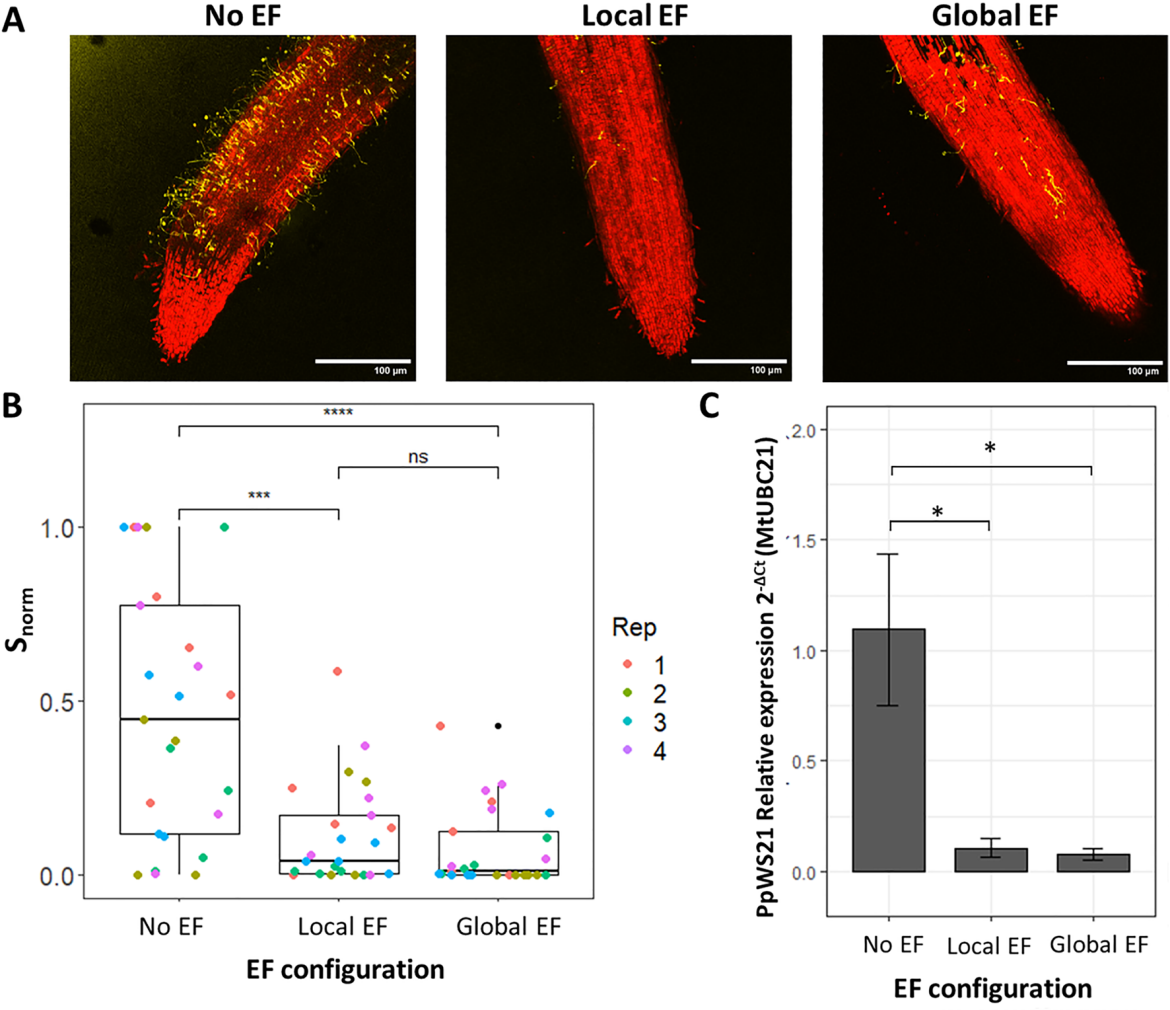
Applying ~ 250 µA reduces P. palmivora infection at different stages in Medicago roots. (**A**) Representative confocal images of roots after 2h infection in control conditions or exposed to 250 µA in the local or global configurations. (**B**) ~ 250 µA applied with a local or global electric field configuration equally reduces zoospore attachment to Medicago roots. Distributions of ${s}_{norm}$ for Medicago roots in the global and local electric field configurations. Each point represents ${s}_{norm}$ for one root; each color represents one technical replicate. *** = *p*-value < 0.001, **** = *p*-value < 0.0001; Wilcoxon test (n = 4 replicates, each with 5 roots). (**C**) Reduction in pathogen biomass after 24h infection measured as relative expression of a pathogen housekeeping gene (ppWS21) compared to a plant housekeeping gene (mtUBC21); * = *p*-value < 0.05; Tukey test (n = 3 replicates, each with 5 roots ).

First, we looked at the effects of ~ 250 μA on zoospore attachment, using the usual assay based on confocal microscopy, and observed a significant reduction in both local and global EF configurations. Interestingly, no difference was detected in this case between the two configurations (Fig. 6A,B).

Since Medicago is a known host for *P. palmivora,* we asked if the same EF intensity would decrease late-stage *P. palmivora* mycelial growth in the root, which is a sign of symptomatic infection^9^. To test this, we exposed the roots to ~ 250 μA in both EF configurations for 24 h, harvested the plant, and extracted mRNA. We quantified the transcripts of the *P. palmivora* housekeeping gene ppWS21 relative to those of the Medicago housekeeping gene mtUBC21, a proxy for pathogen biomass^23^. Interestingly, in this case, we observed a significant decrease in pathogen biomass (Fig. 6C) in both EF configurations.

Finally, to confirm that long exposure to the local EF configuration in soil (the mock root) does not alter plant health, we exposed Medicago roots to ~ 250 μA in the local EF configuration (mock root) in soil for over 14 days and measured leaf number and the whole plant’s dry and fresh weights: as with Arabidopsis, no statistically significant difference was observed between exposed and not-exposed plants (Supplementary Fig. 6).

Overall, this data indicates that external EFs are effective at reducing *P. palmivora* zoospore attachment and biomass in Medicago roots, without affecting plant health.

## Discussion

In this paper, we provide experimental evidence that external EFs can effectively mitigate root infections caused by the oomycete pathogen *P. palmivora.* The work is based on the hypothesis that EFs can disrupt the bioelectric interactions between host and pathogen. Overall, our data indicate that external EFs can curtail the early stages of infection. Notably, immersing roots in a “global” configuration of ionic currents can be more effective, as shown here in Arabidopsis, compared to using “local” currents (“mock root” configuration) positioned near but not across the roots.

This is an unexpected and interesting result that requires some interpretation. We had previously shown that ionic currents are more important than nominal EFs in zoospore electrotactic response^12^, but we had not explored the role of current density (i.e. current intensity divided by the surface area of the electrodes, or the current going through a unit area). We notice that the current densities in our two configurations are different (Table 1), but this alone is not sufficient to explain differences in protection efficiency, since both configurations result in similar zoospore attachment inhibition in Medicago roots (Fig. 6B).

**Figure 7:**
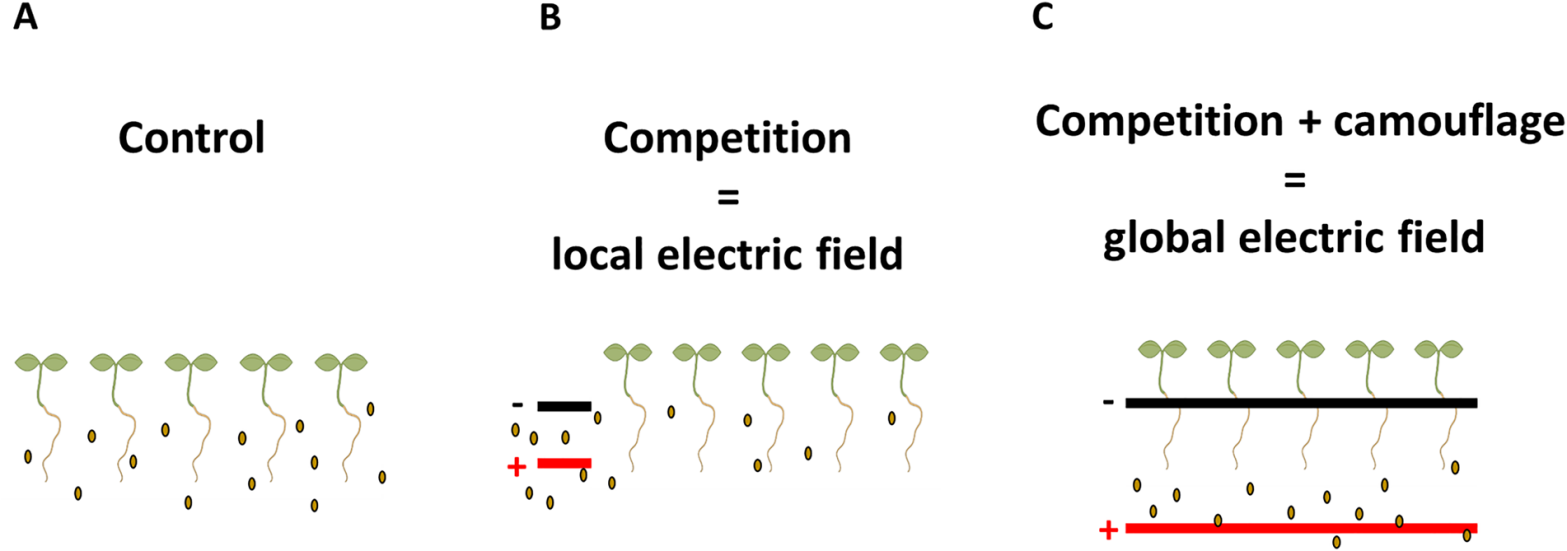
Model of global and local EF mode of action. (**A**) In the absence of an external EF, the zoospores are uniformly attracted to the plant roots in the medium. (**B**) When exposed to a local EF, zoospores are more attracted to the artificial root than the plant roots. The system acts as a competition assay. (**C**) When exposed to a global EF, zoospores are attracted to the positive electrode, which is further away from the roots, thus acting as competition. However, by generating an electrical current, the global EF is also dispersing the plant's endogenous electric signature. Therefore, the global EF provides competition and camouflage.

We propose two plausible, not mutually exclusive, mechanisms to explain the action of external EFs in these experiments (Fig. 7). In the first mechanism, operating in both local and global configurations (Fig. 7B,C), the applied EF competes with the root endogenous bioelectric field^25^ to attract the zoospores via their natural electrotropic response. Since the endogenous EFs measured in roots are generally quite weak, generating current densities ranging between 0.002 µA/mm^2^ in Ryegrass (*Lolium perenne*) and 0.027 µA/mm^2^ in Peanut (*Arachis hypogaea*)^25^, the stronger current densities generated by the artificial EFs in this work (Table 2) seem likely to win such competition and to drive the zoospores away from the natural roots. In the second mechanism, only active in the global configuration (Fig. 7B), the applied EF overlaps and perturbs the root natural ionic currents, effectively “camouflaging” them and making them undetectable by the zoospores.

Given this model, Medicago roots might be equally protected by the two EF configurations for two reasons. First, Medicago root’s bioelectric field and ionic currents might be stronger than the ones in Arabidopsis, making it more difficult to camouflage and therefore making the global configuration less effective than with Arabidopsis. However, this is unlikely as the highest endogenous current density recorded to date (0.027 μA/mm^2^ in Peanut^13^) is 20 times smaller than the one generated by our global EF (0.4 ± 0.02 μA/mm^2^). Second, near Medicago roots, the zoospores might exhibit stronger chemotaxis (i.e. movement along chemical gradients) than electrotaxis (i.e. movement along electric fields and ionic currents). Indeed, *P. palmivora* zoospores have been shown to respond strongly to chemical gradients, especially those generated by root exudates of host species^26–28^. This would make the camouflaging of the root’s endogenous bioelectric field irrelevant, therefore making the effect of local and global EFs comparable.

We have also shown that both configurations of external EFs reduce *P. palmivora* biomass in Medicago’s roots (Fig. 6C). Although this is a valuable independent confirmation, based on molecular data, of our confocal imaging, this is not sufficient to conclude that external EFs also decreased late mycelial growth. In fact, the reduction in *P. palmivora* biomass could be explained simply by the reduction in zoospore attachment (Fig. 6B). Interestingly, external EFs are known to affect hyphal growth and direction in fungi^29–31^ as well as the directionality of the germ tube growth toward a positive charge in *P. palmivora*^12^. Thus, we can speculate that an external EF with the positive electrode located below the root tip might keep the mycelial growth confined in the root, preventing its spread to the rest of the plant tissue. Future work based on live imaging of hyphae distribution within the root tissue might test the hypothesis.

The long-term goal of research in plant–pathogen interactions is to uncover novel biological mechanisms that might be used to develop more effective crop protection strategies. Ongoing research is primarily directed toward understanding the molecular mechanisms underlying plant resistance to *Phytophthora* and developing genetically modified crops. However, it's essential to note that this cannot be achieved rapidly and does not adequately address the swift evolution of pathogen resistance avoidance genes^32^. By studying the bioelectric component of host–pathogen interaction at its most fundamental levels, we have the opportunity to develop new methods for crop protection.

Here, we are proposing to use artificial EFs to divert zoospores away from plant roots, providing an alternative preventative strategy that relies on a response to a physical stimulus and that can be used in combination with other methods to minimize the pathogen burden. Within the scope of *P. palmivora’s* natural hosts, mostly plantation crops^4,5^, we believe the local EF configuration is a more applicable tool as a mock root could be placed at the most protective distance from the plant root system. Crucially, we showed that the external EFs did not have an impact on the biomass and leaf number of Arabidopsis and Medicago (Supplementary Fig. 6). We only observed a small but significant reduction in primary root growth in Arabidopsis, but this did not impair root gravitropism (Fig. 5). This suggests that external EFs comparable to those discussed in this paper will not negatively impact plant health.

Overall, we showed that weak external EFs can be used to reduce *P. palmivora* zoospore infection in hydroponics without damaging the plant. Future work should focus on investigating this phenomenon in soil and host species like cocoa, papaya, and oil palm.

## Materials and methods

### *P. palmivora* culture conditions

*P. palmivora* YFP (P16830 LILI YFP KDEL) mycelia were cultured on V8 medium (100 ml/l V8 juice, 1 g/l CaCO_2_, 50 mg/l *β*-sistosterol, 15 g/l bacteriological agar, 50 μg/ml G418) at 28 °C in constant light. The zoospores were released and collected by flooding a 6 d-old plate with 5 ml of 1/500X liquid MS medium (1X MS medium is 5 g/l sucrose, 8.6 mg/l Murashige and Skoog (MS) salts, 0.5 g/l MES hydrate buffer, adjusted to pH 5.7 with 1 M Tris HCL) and incubating for 1 h at 22 °C. After collection, the zoospores were counted using a hemocytometer, and the solution was diluted with MS media to the final concentration of 50,000 zoospores/ml.

### Plant growth and culture conditions

In this paper, we used research lines for the plant model systems: wild-type *Arabidopsis thaliana* (Col-0), obtained from the Nottingham Arabidopsis Stock Centre (N1092), and wild-type *Medicago truncatula* (Jemalong A17), donated by Prof. C. Turnbull. No wild plant specimens were collected from the field in this work, and all methods were performed following relevant guidelines. Arabidopsis seeds were imbibed in water and kept in the dark for 2 days at 4 °C to synchronize germination. All seeds were surface sterilized using 50% (v/v) Haychlor bleach and 0.0005% (v/v) Triton X-100 for 3 min and then rinsed 6 times with sterilized Milli-Q water. Seeds were germinated and grown in nurseries as described in^21^. Briefly, seeds were sown individually inside PCR tubes filled with 1X MS gel medium: 0.44% (w/v) Murashige and Skoog (MS) Basal medium (Sigma-Aldrich, M5519), 0.5% (w/v) sucrose, 0.05% (w/v) MES hydrate (Sigma-Aldrich M8250), 0.8% (w/v) agar (Sigma-Aldrich 05,040), pH adjusted to 5.7 with TRIS HCl (Fisher-Scientific 10,205,100). The PCR tubes had their end cut out to allow the root to grow through and placed in a 3D-printed (Ultimaker 2 +) holder inserted in a Magenta box (Sigma-Aldrich V8380). The Magenta box was filled with 150 ml of 1/500X MS liquid medium (0.00088% (w/v) MS Basal medium, 0.5% (w/v) sucrose, 0.05% (w/v) MES hydrate, pH5 adjusted to 5.7 with TRIS HCl) to reach the end of the PCR tubes. These “germination” or “nursery” boxes were placed in a growth chamber at 23 °C, with a 16 h / 8 h light/dark photoperiod and light intensity of 120 µmol/m^2^ s.

For *Medicago truncatula* (A17)*,* seeds underwent initial scarification using sandpaper, followed by a thorough 90-s wash with 12% bleach. The treated seeds were subsequently rinsed six times with sterilized Milli-Q water. After this process, the seeds were transferred onto plates with 1X MS solid (0.8% (w/v) agar) medium and kept in darkness at 4°C for three days. The seeds on plates were then moved to 22°C with 16 h / 8 h light/dark photoperiod and light intensity of 120 µmol/m^2^ s.

All experiments were conducted with primary roots of seedlings 5–8 days post-germination.

### Design and fabrication of the V-box

The setup used in this work was a modified version of the V-box previously described^21^, to generate a vertical rather than horizontal EF. Design and manufacturing of both the V-box and the mock root were performed using the CAD software OnShape and an Ultimaker 2 + 3D printer with PLA filament.

For the global EF configuration, three platinum–iridium foils with 5 perforations were slotted in the top electrode compartment, and three more were clipped in the bottom one with an *ad-hoc* clip (Fig. 1A and Supplementary Fig. 1 and Supplementary Fig. 2). For the local EF configuration, two platinum–iridium foils were cut to size and slotted in the two compartments of the 3D-printed mock root (Fig. 1C and Supplementary Fig. 3). The mock root was then inserted in the V-box for the local EF (Fig. 1B and Supplementary Fig. 4). The system was enclosed in a glass jar containing 2 l of 1/500X MS liquid medium and wired to a PS-1302 D power supply (Voltcraft, UK).

### Electric field tolerance assay

For the root EF tolerance assay in hydroponics, each PCR tube containing a single 7 to 10-day-old seedling was transferred to the V-box. The plantlets were then exposed to 250 µA for 24 h and transferred to 1X MS solid (0.8% (w/v) agar) medium plates placed vertically. Images were taken 48 h later and root growth and gravitropic response angles were measured using ImageJ.

For the root electric field tolerance assay in soil, Arabidopsis and Medicago were transplanted to the soil-vermiculate mixture (soil:vermiculite = 3:1) in pots with 10 cm × 10 cm soil surface. Arabidopsis seedlings were placed in groups of 16 per pot, while Medicago seedlings were placed in groups of 7 per pot. Mock roots were inserted into the corners of the pots, approximately 1–15 cm away from any given plant. Following a two-week growth period, data were collected for leaf count, and whole plant fresh and dry weight. Dry weight was determined through freeze drying.

### Root infection assay and confocal microscopy

For infection assays, each PCR tube containing a single 7 to 10-day-old seedling was transferred to the V-box and 1 ml of zoospore solution (50,000 zoospores/ml) was pipetted after the electric field was turned on. The roots were then removed after 2 h for live confocal imaging or 24 h for biomass quantification.

Infected plantlets were mounted in water on standard microscope slides. Imaging was performed with a Leica TCS SP5 resonant inverted confocal microscope, with 10 × dry and 63 × water immersion lenses. The number of zoospores attached to the root was counted using the cell counter tool in ImageJ software and we calculated ${s}_{norm}$ as follows:

$${s}_{norm}= \frac{{s}_{r}}{{s}_{max}}$$

where ${s}_{r}$ is the number of zoospores attached to the root of interest with $r=\{1;2;3;4;5\}$ and ${s}_{max}$ is the maximum number of zoospores attached to a root within the same technical repeat.

### RNA extraction and qPCR quantification

Pathogen biomass quantification was performed using RT-qPCR with the method described^23^. For both Arabidopsis and Medicago, the infected sample containing 5 plantlets was snap-frozen in liquid nitrogen. The tissue was ground into a fine powder and RNA was extracted using the RNaeasy Plus Mini Kit (Quiagen). This was followed by DNAse treatment with the TURBO DNA-free™ Kit (Thermo Fisher). The quality of the RNA was assessed with Nanodrop and running on a 1% agarose gel. The Transcriptor First Strand cDNA Synthesis Kit (Roche) was used to synthesize first-strand cDNA.

For the RT-qPCR we prepared a master mix containing 0.4 μl forward primer, 0.4 μl reverse primer, 10 μl 1X Fast Sybr Green 2X (Thermo Fisher), and 7.2 μl of nuclease-free water per reaction. 18 μl of stock solution was then added to each well of a 96-well plate (Thermo Fisher) together with 2 μl of cDNA. qPCR of 55 cycles at annealing temperature of 60 °C, melting temperature of 90 °C and elongation temperature of 72 °C was performed using the LightCycler® 480 II (Diagnostics, 2008) and the LightCycler480 software (Version 1.5.1.62 SP2) was used to analyze the data. Following established methods, the *P. palmivora* gene (ppWS21) was normalized to the Arabidopsis and Medicago housekeeping genes atUBC21 and mtUBC21, respectively, using the 2^−ΔCt^ method^33^. All primer sequences can be found in Supplementary Table 1.

### Supplementary Information

Supplementary Information.

## Data Availability

The raw data that support the findings of this study are available from the corresponding author upon reasonable request.
