# Supplementary material for: Reduction of Phytophthora palmivora plant root infection in weak electric fields
Source: Sci Rep. 2024 Aug 28;14:19993. doi: 10.1038/s41598-024-68730-y (PMC11358279; doi:10.1038/s41598-024-68730-y)
Supplement: Supplementary file 1 — Supplementary Information. [file 41598_2024_68730_MOESM1_ESM.pdf]

Reduction of *Phytophthora palmivora* plant root infection in weak electric fields

Eleonora Moratto[1], Zhengxi Tang[1], Tolga O. Bozkurt[1], Giovanni Sena[1]*

[1]Department of Life Sciences, Imperial College London, UK
Corresponding author
g.sena@imperial.ac.uk

Supplementary Information

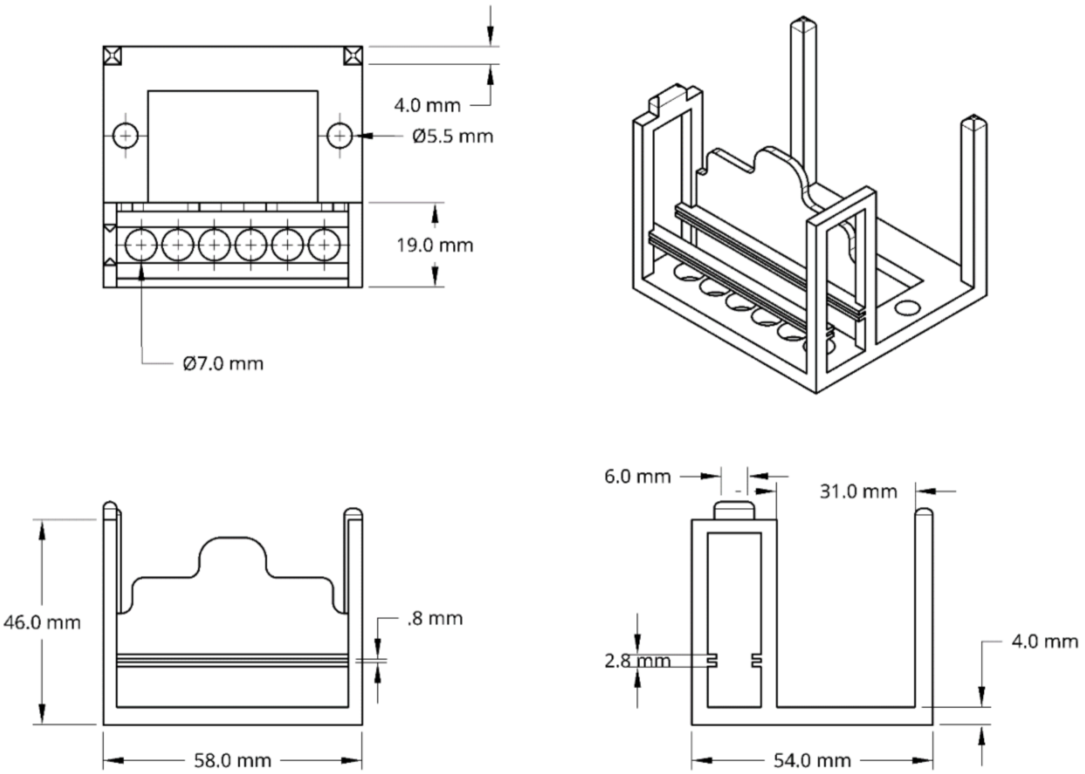

***Supplementary Figure 1**:* Summary of the CAD schematic used to 3D print the V-box for global electric field configuration.

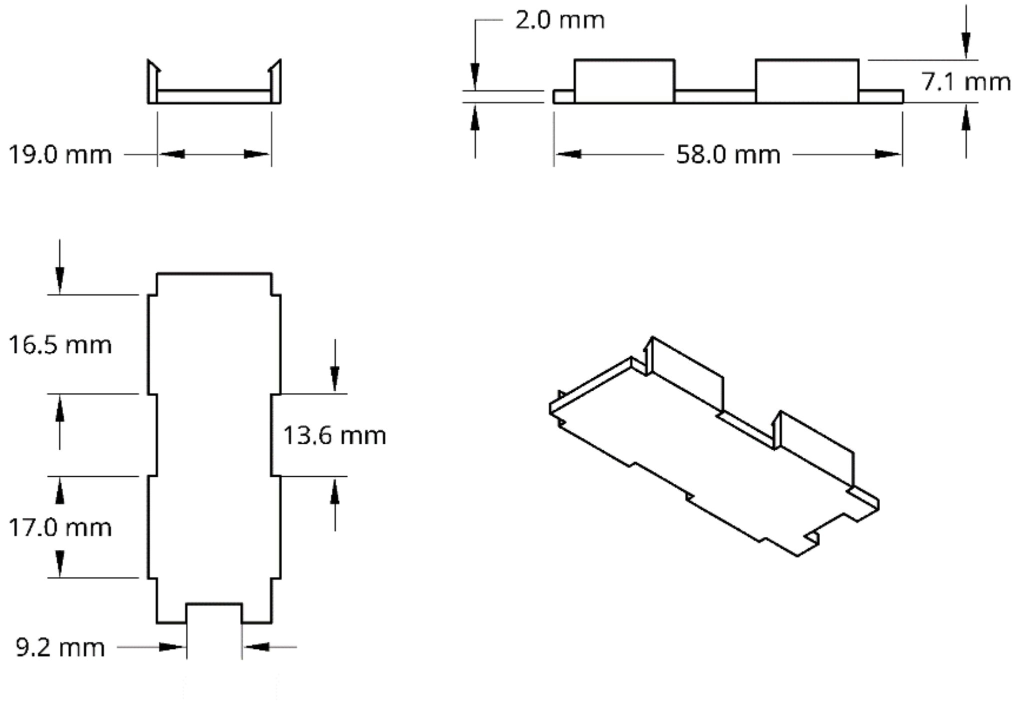

Supplementary Figure 2*:* Summary of the CAD schematic used to 3D print the foil clip that holds the bottom electrodes in the V-box for global electric field configuration.

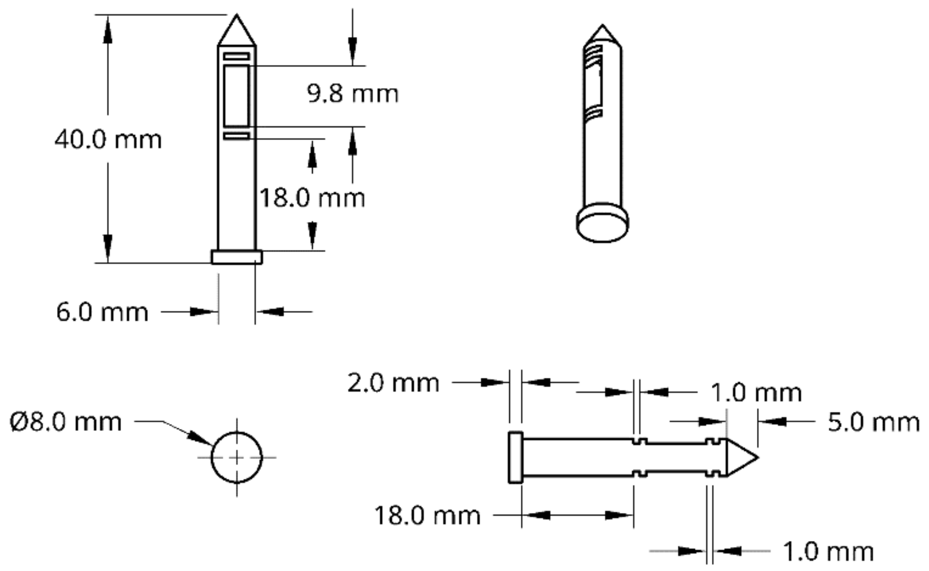

Supplementary Figure 3*:* Summary of the CAD schematic used to 3D print the mock root.

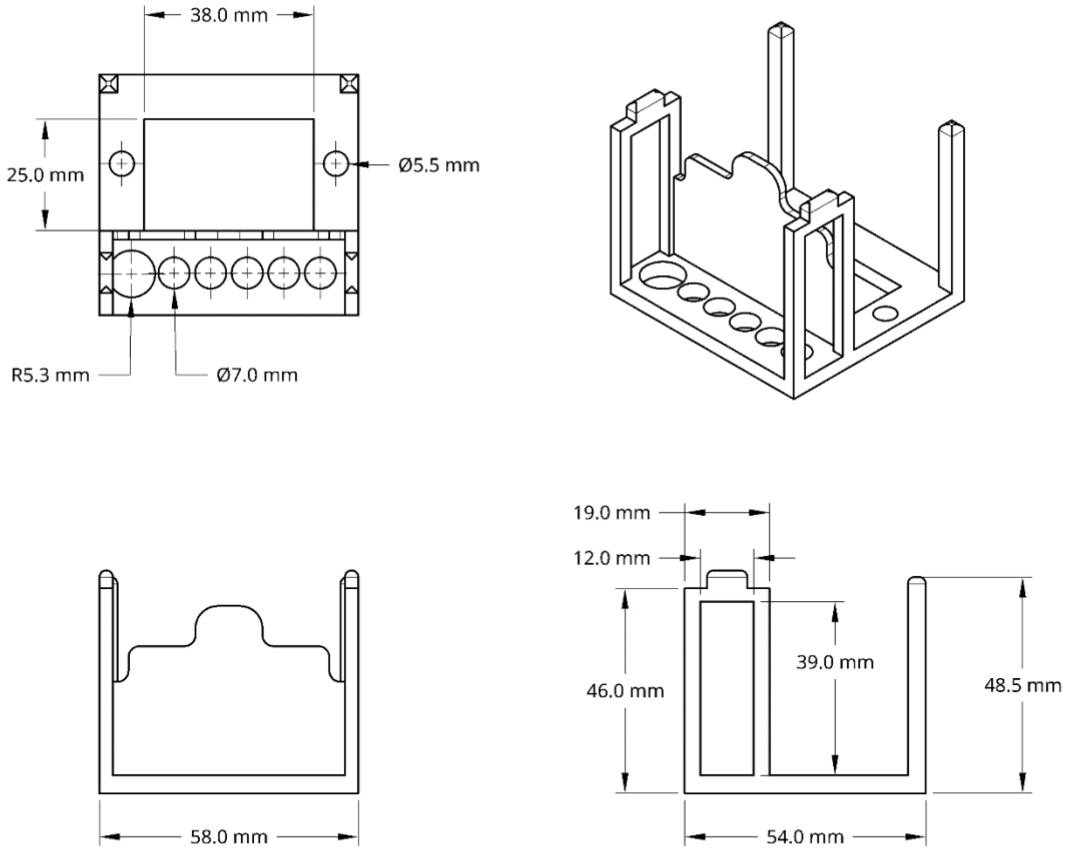

***Supplementary Figure 4**:* Summary of the CAD schematic used to 3D print the V-box for local electric field configuration.

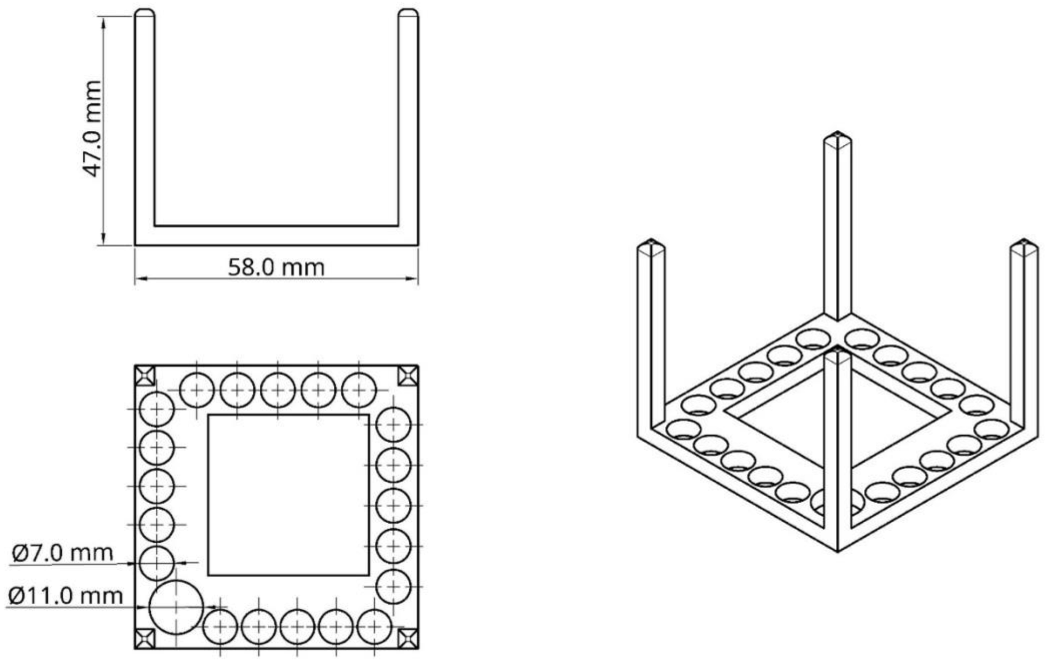

***Supplementary Figure 5**:* Schematic of the 3D-printed V-box set-up to assess the radius effect of the local EF on root infection assays with P. palmivora zoospores.

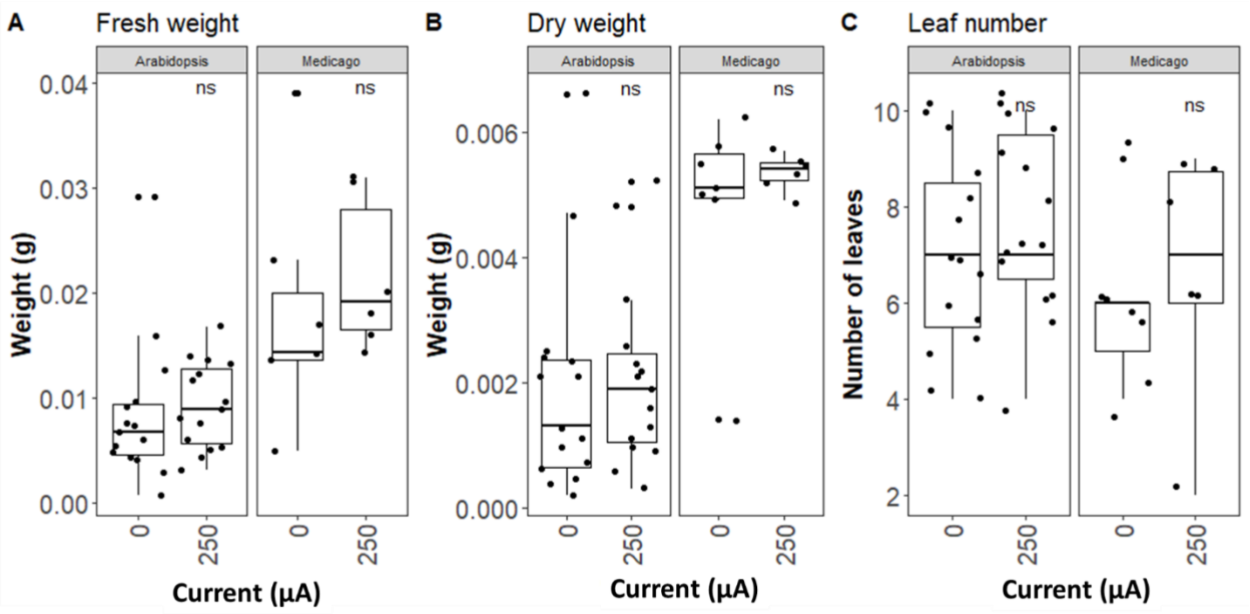

Supplementary Figure 6*:* 14-day exposure to 250 μA current generated by a local electric field does not affect fresh weight, dry weight, or leaf number. Distribution of fresh weight A), dry weight B), and leaf number B) of Arabidopsis (left panels) and Medicago (right panels) seedlings exposed to 250 μA produced by a local electric field in soil for 14 days. Each point represents one root. (ns = p-value > 0.05; Wilcoxon rank sum exact test (n = 15)).

**Primer name**	**Primer sequence**	**Organism**
AtUBC21_qF	TCCTCTTAACTGCGACTCAG	*Arabidopsis thaliana*
AtUBC21_qR	GCGAGGCGTGTATACATTT	*Arabidopsis thaliana*
MtUBC21_qF	GCAGATAGACACGCTGGGA	*Medicago truncatula*
MtUBC21_qR	AACTCTTGGGCAGGCAATAA	*Medicago truncatula*
PpWS21_qF	CTCCAGAACGTGTACATTCG	*Phytophthora palmivora*
PpWS21_qR	TGGCACCCTTCTCCTCGG	*Phytophthora palmivora*

Supplementary Table 1: Primers used for RT-qPCR in the infection assay.
